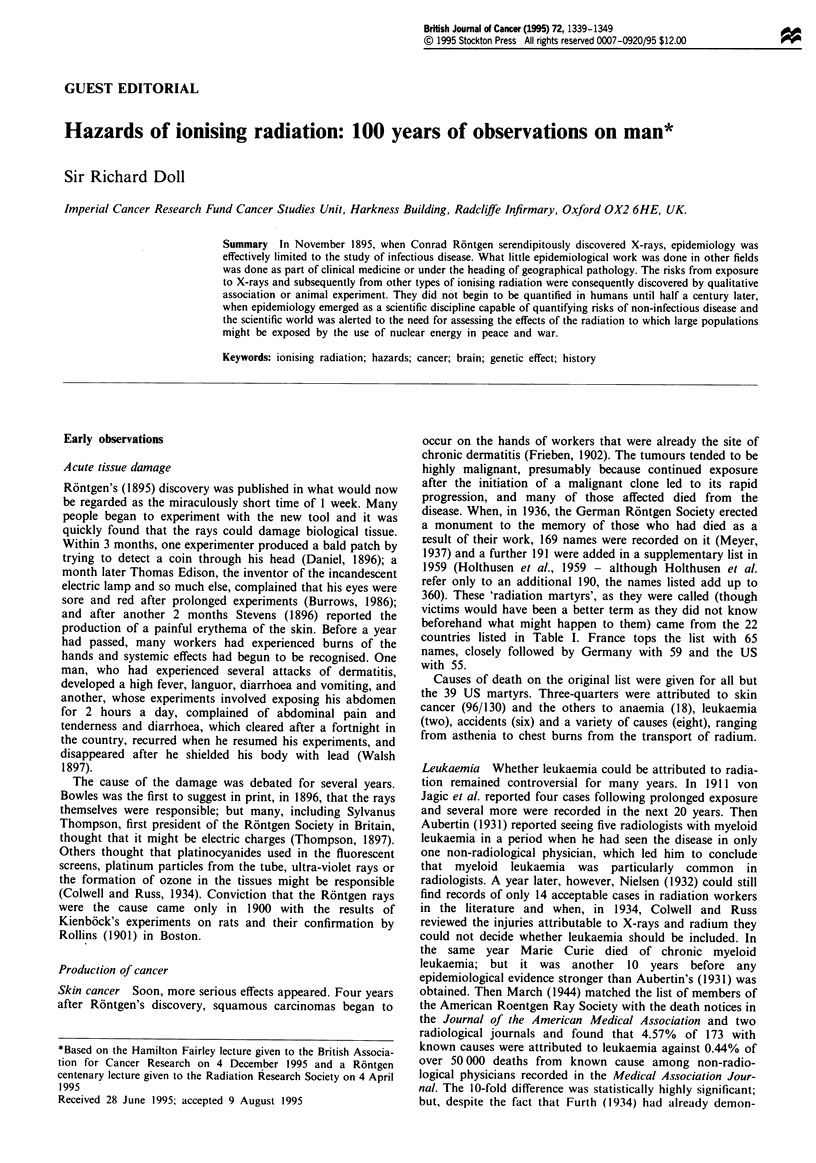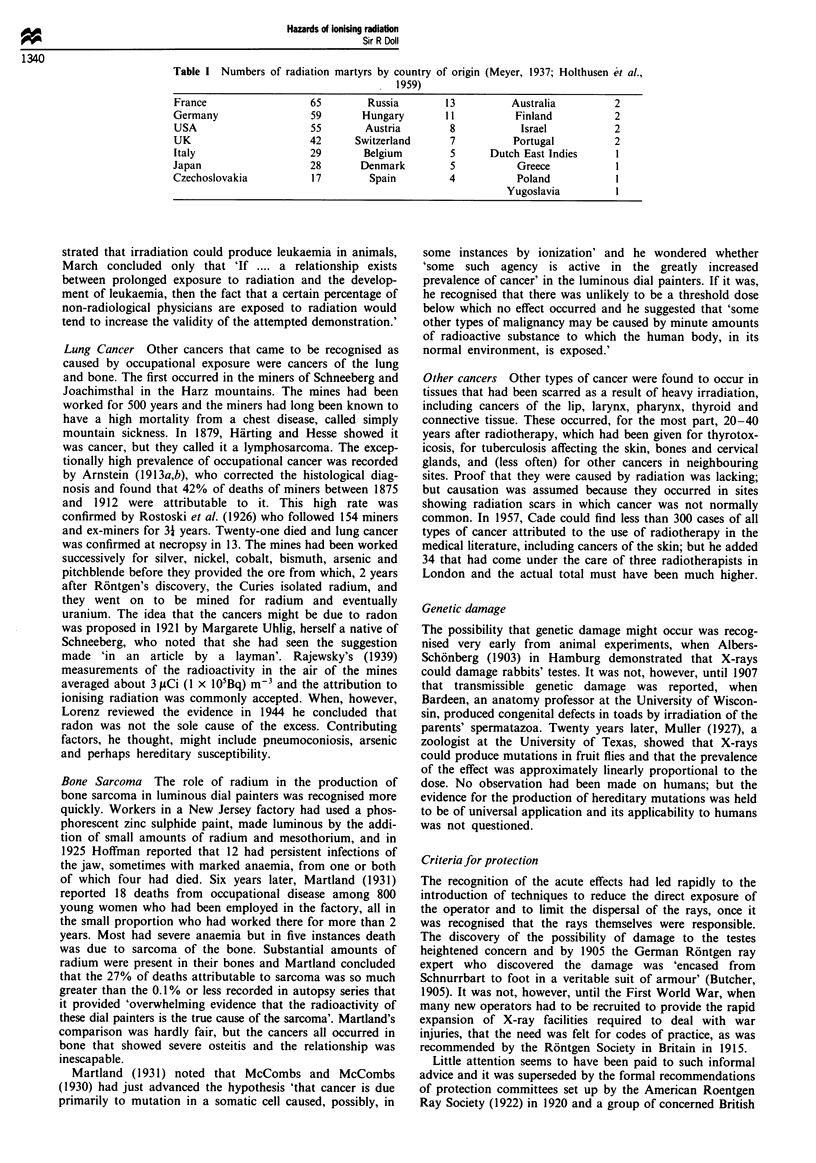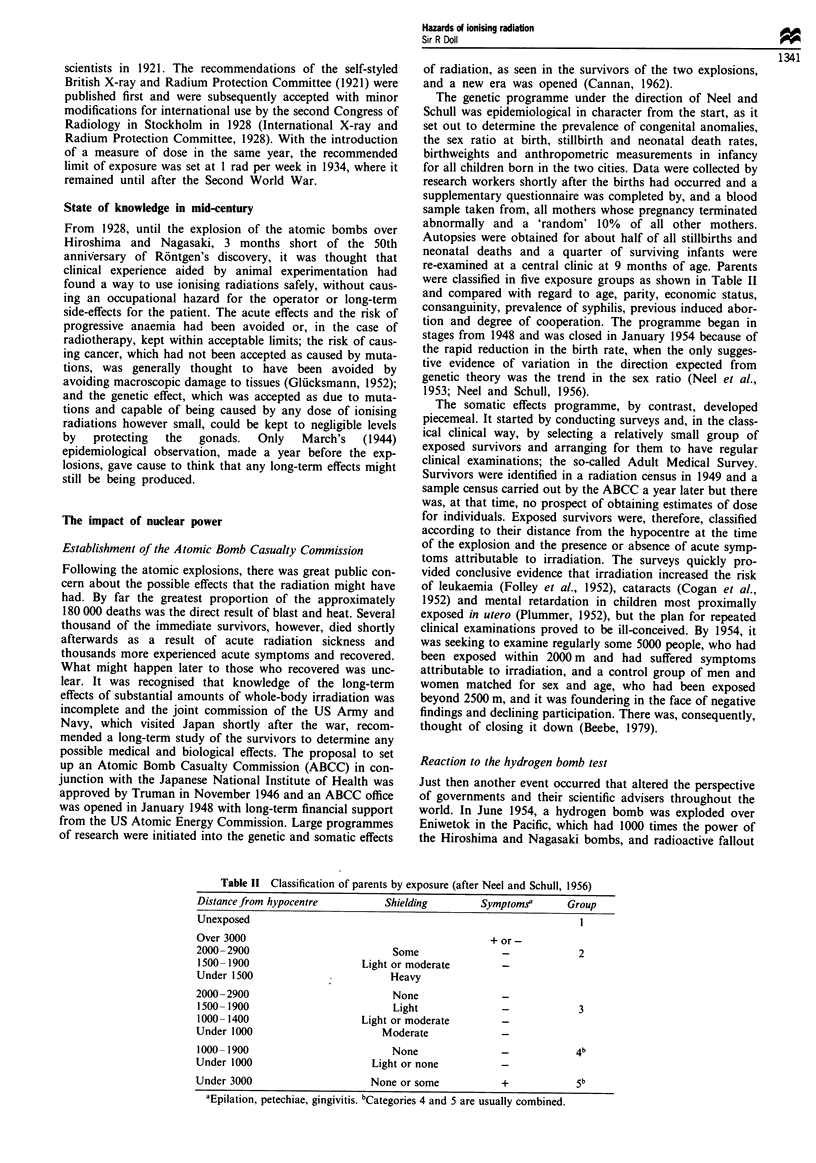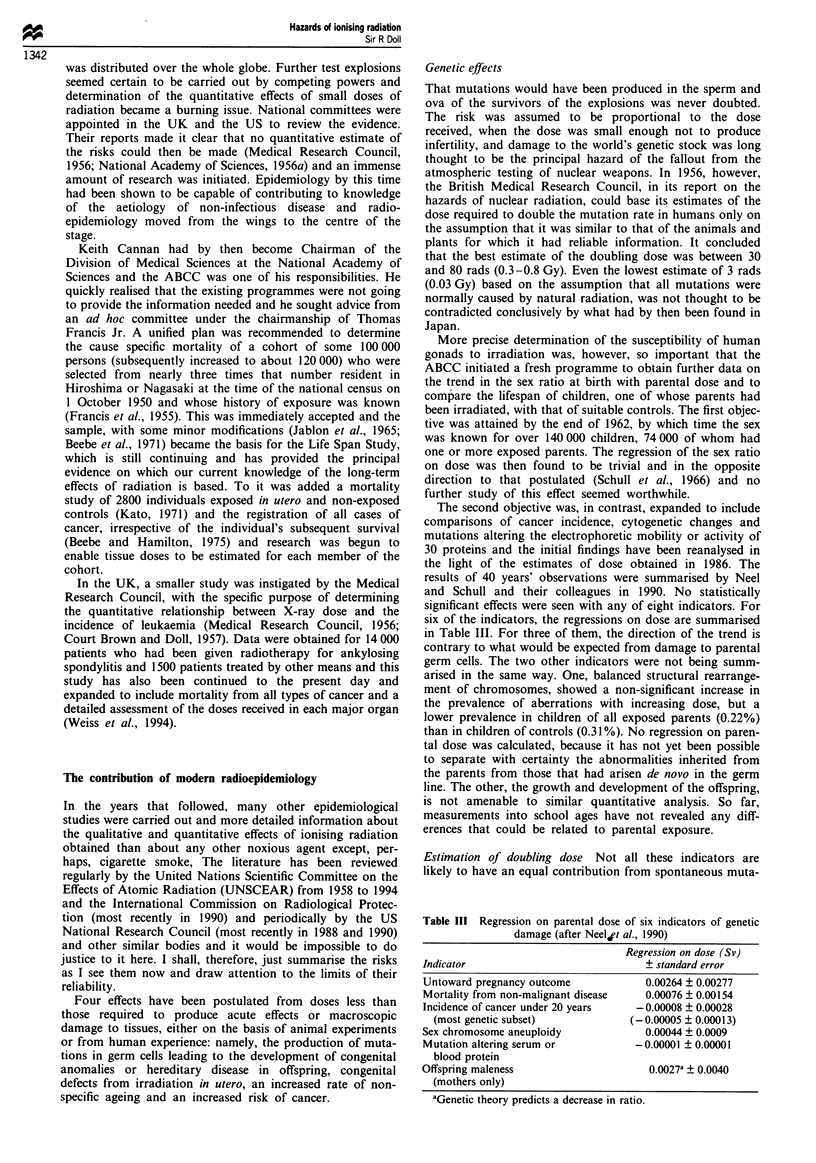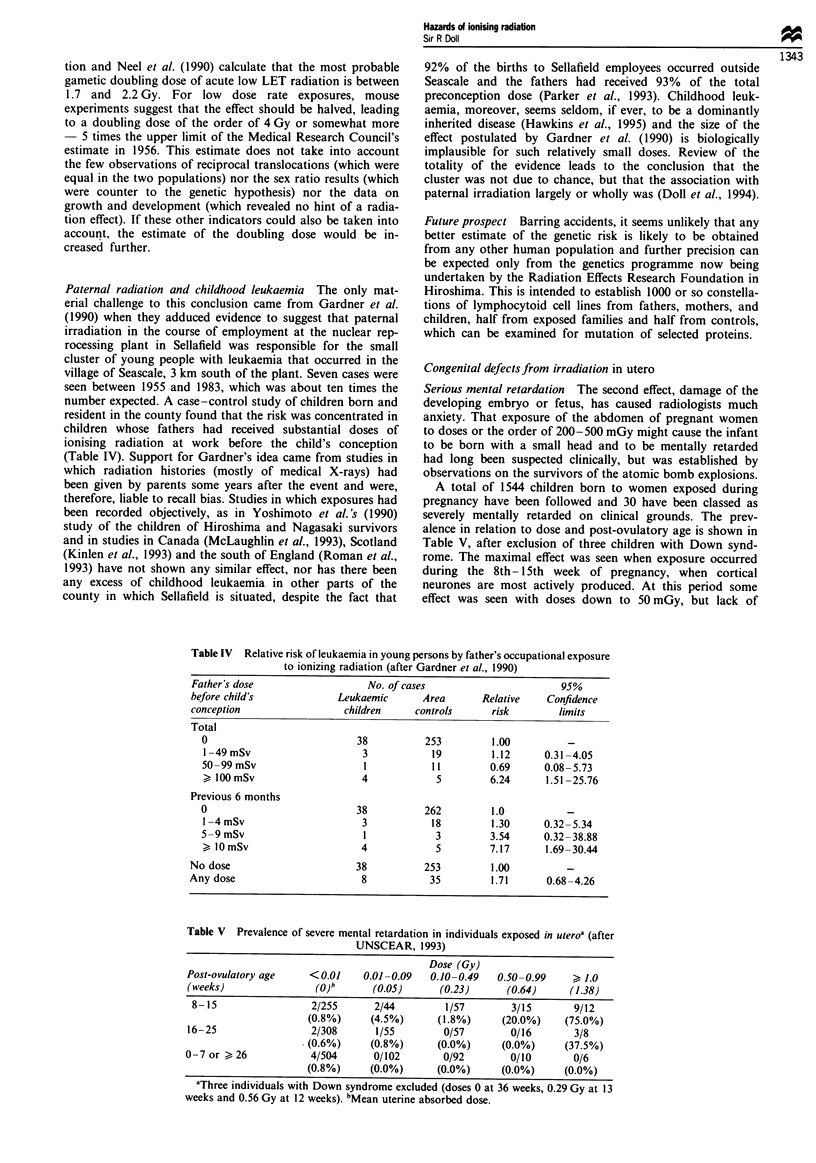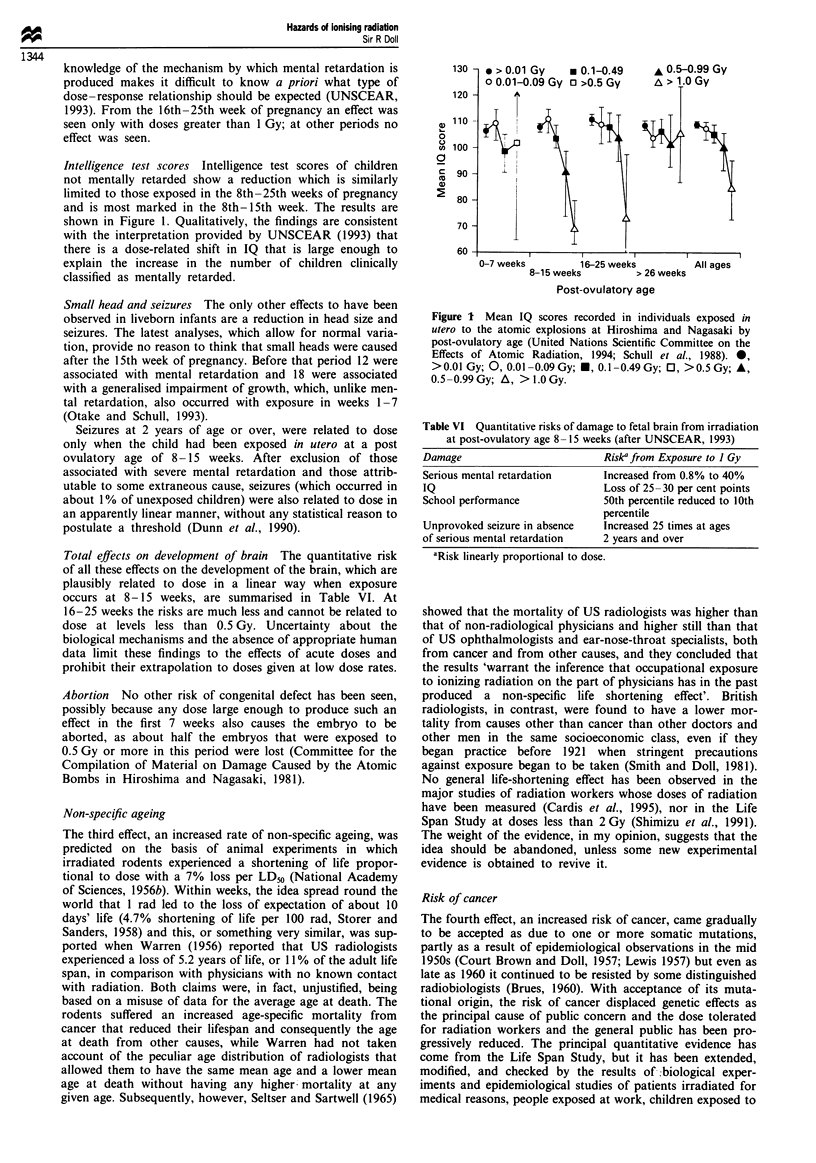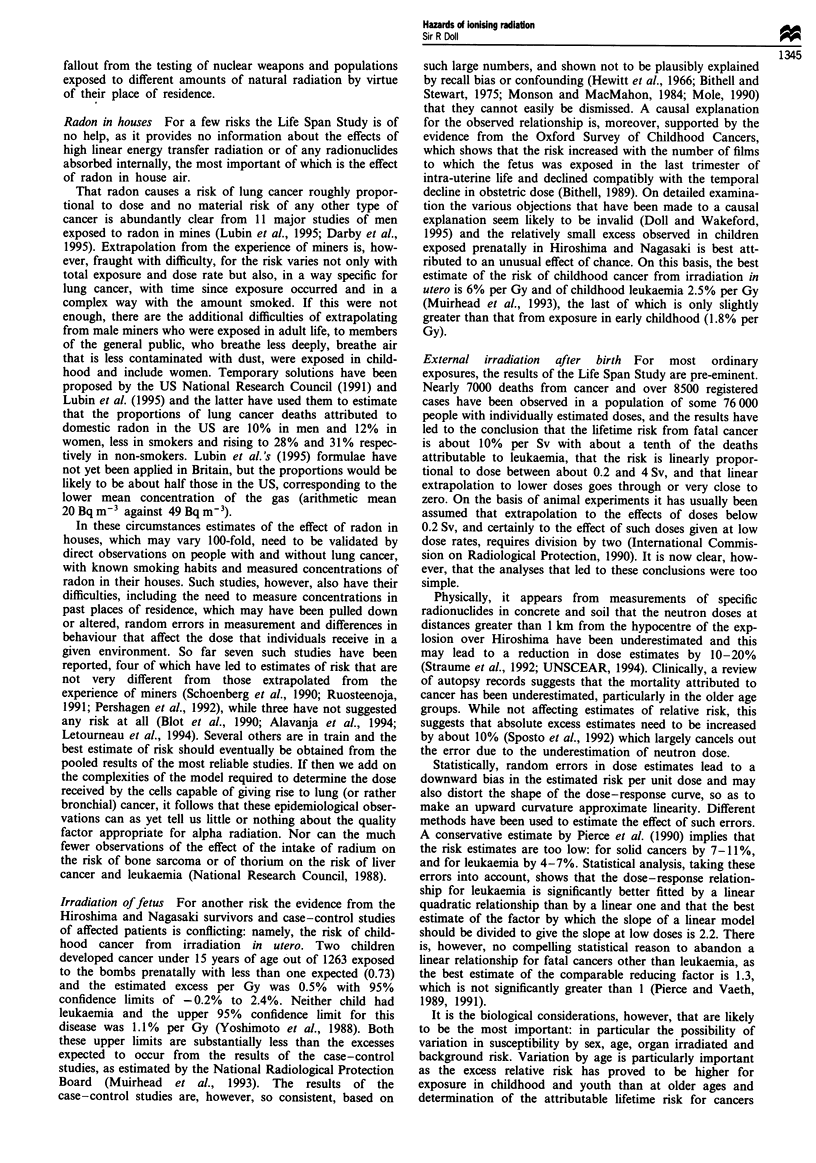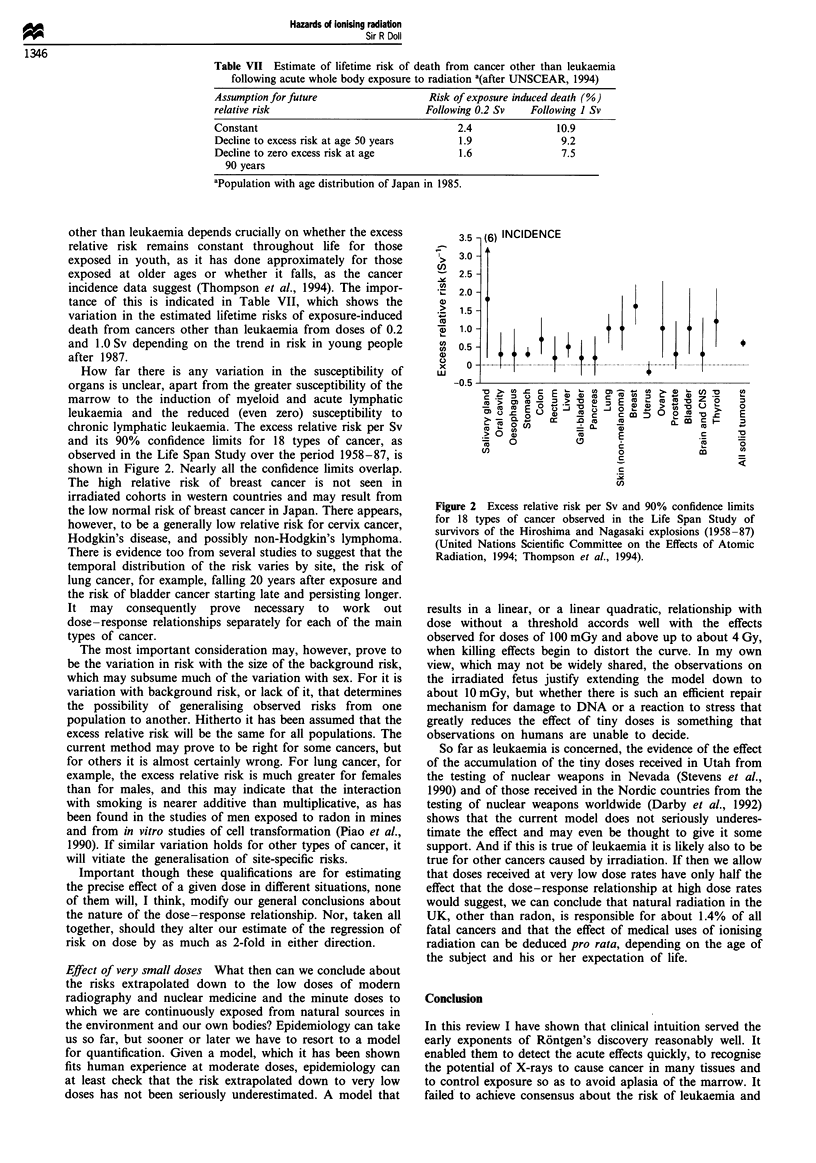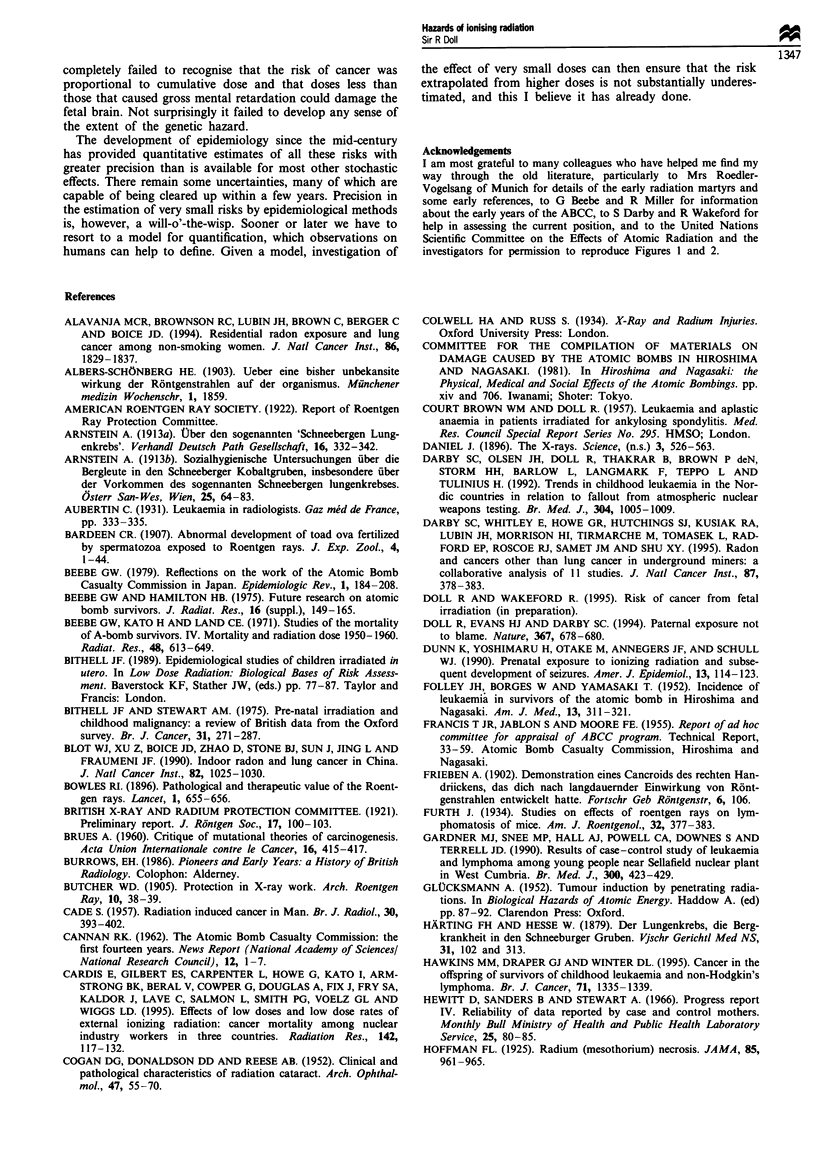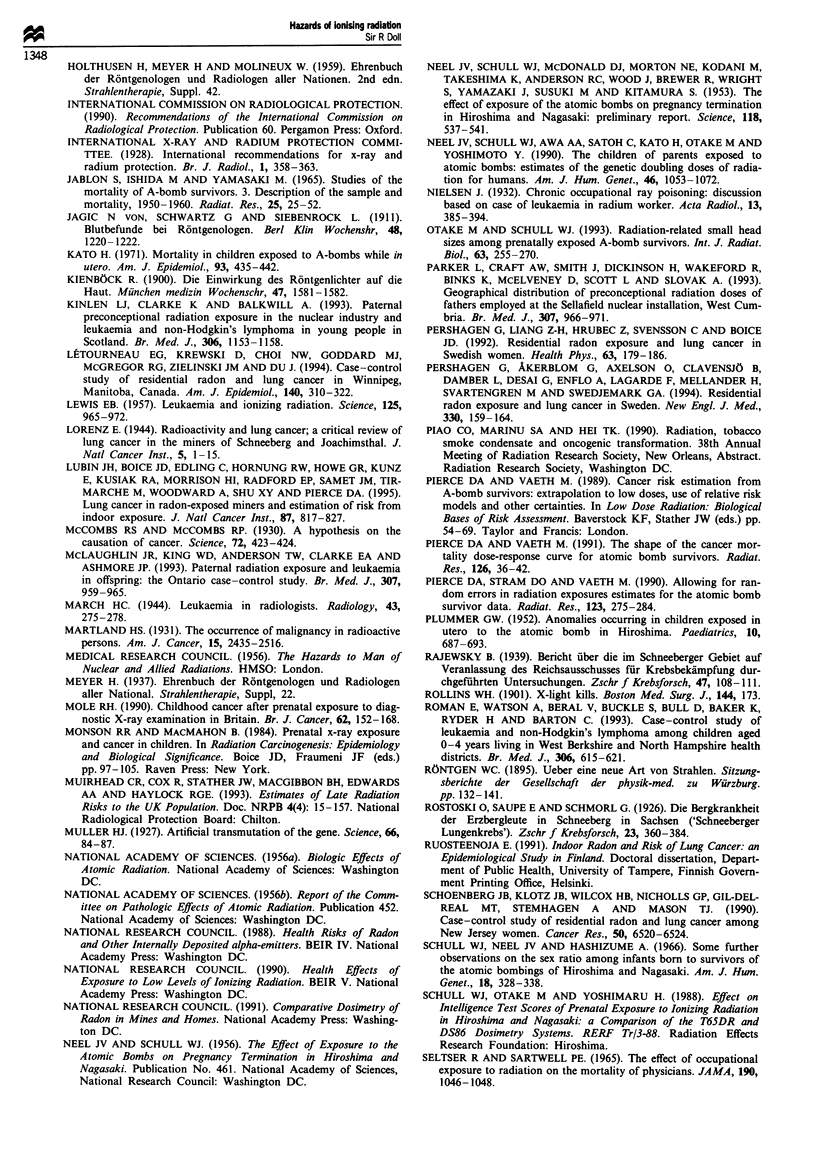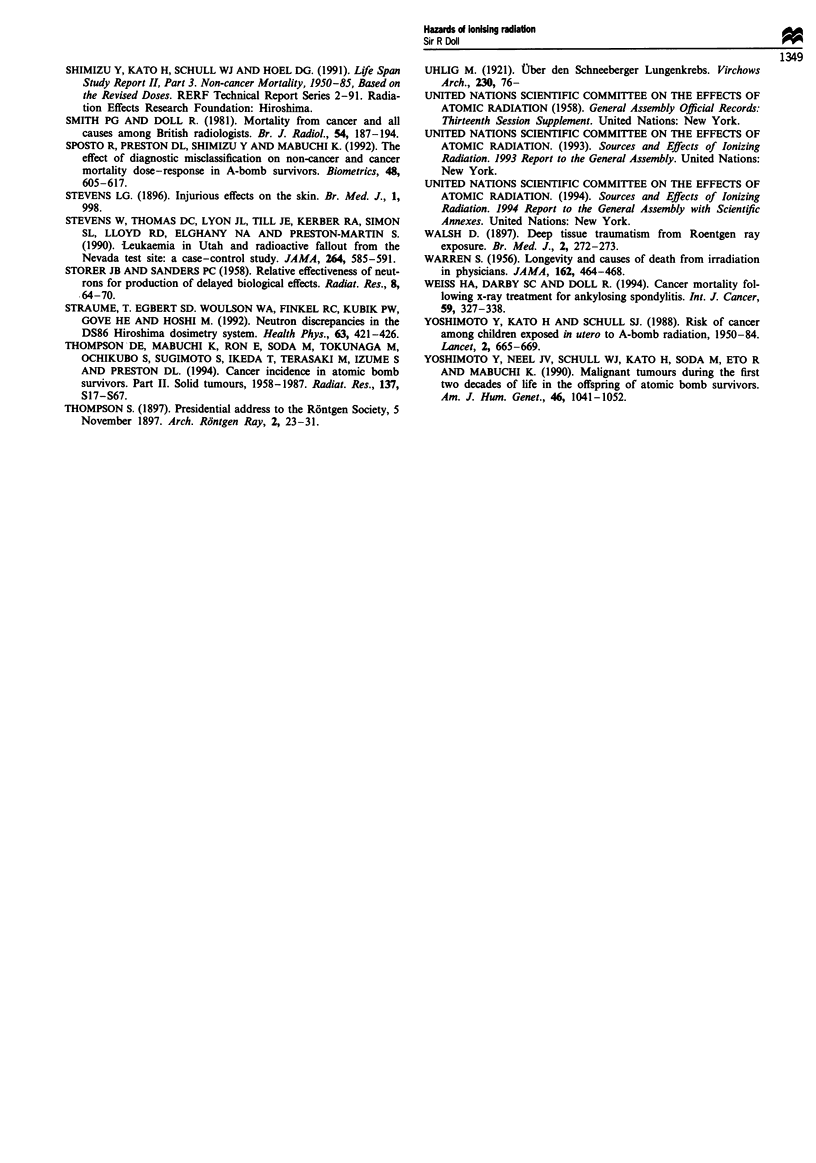# Hazards of ionising radiation: 100 years of observations on man.

**DOI:** 10.1038/bjc.1995.513

**Published:** 1995-12

**Authors:** R. Doll

## Abstract

In November 1895, when Conrad Röntgen serendipitously discovered X-rays, epidemiology was effectively limited to the study of infectious disease. What little epidemiological work was done in other fields was done as part of clinical medicine or under the heading of geographical pathology. The risks from exposure to X-rays and subsequently from other types of ionising radiation were consequently discovered by qualitative association or animal experiment. They did not begin to be quantified in humans until half a century later, when epidemiology emerged as a scientific discipline capable of quantifying risks of non-infectious disease and the scientific world was alerted to the need for assessing the effects of the radiation to which large populations might be exposed by the use of nuclear energy in peace and war.


British Journal of Cancer (1995) 72, 1339-1349
© 1995 Stockton Press All rights reserved 0007-0920/95 $12.00

GUEST EDITORIAL

### Hazards of ionising radiation: 100 years of observations on man*

Sir Richard Doll

*Imperial Cancer Research Fund Cancer Studies Unit, Harkness Building, Radcliffe Infirmary, Oxford OX2 6HE, UK.*

**Summary** In November 1895, when Conrad Röntgen serendipitously discovered X-rays, epidemiology was effectively limited to the study of infectious disease. What little epidemiological work was done in other fields was done as part of clinical medicine or under the heading of geographical pathology. The risks from exposure to X-rays and subsequently from other types of ionising radiation were consequently discovered by qualitative association or animal experiment. They did not begin to be quantified in humans until half a century later, when epidemiology emerged as a scientific discipline capable of quantifying risks of non-infectious disease and the scientific world was alerted to the need for assessing the effects of the radiation to which large populations might be exposed by the use of nuclear energy in peace and war.

**Keywords:** ionising radiation; hazards; cancer; brain; genetic effect; history

### Early observations

### *Acute tissue damage*

Röntgen's (1895) discovery was published in what would now be regarded as the miraculously short time of 1 week. Many people began to experiment with the new tool and it was quickly found that the rays could damage biological tissue. Within 3 months, one experimenter produced a bald patch by trying to detect a coin through his head (Daniel, 1896); a month later Thomas Edison, the inventor of the incandescent electric lamp and so much else, complained that his eyes were sore and red after prolonged experiments (Burrows, 1986); and after another 2 months Stevens (1896) reported the production of a painful erythema of the skin. Before a year had passed, many workers had experienced burns of the hands and systemic effects had begun to be recognised. One man, who had experienced several attacks of dermatitis, developed a high fever, languor, diarrhoea and vomiting, and another, whose experiments involved exposing his abdomen for 2 hours a day, complained of abdominal pain and tenderness and diarrhoea, which cleared after a fortnight in the country, recurred when he resumed his experiments, and disappeared after he shielded his body with lead (Walsh 1897).

The cause of the damage was debated for several years. Bowles was the first to suggest in print, in 1896, that the rays themselves were responsible; but many, including Sylvanus Thompson, first president of the Röntgen Society in Britain, thought that it might be electric charges (Thompson, 1897). Others thought that platinocyanides used in the fluorescent screens, platinum particles from the tube, ultra-violet rays or the formation of ozone in the tissues might be responsible (Colwell and Russ, 1934). Conviction that the Röntgen rays were the cause came only in 1900 with the results of Kienböck's experiments on rats and their confirmation by Rollins (1901) in Boston.

### *Production of cancer*

*Skin cancer* Soon, more serious effects appeared. Four years after Röntgen's discovery, squamous carcinomas began to occur on the hands of workers that were already the site of chronic dermatitis (Frieben, 1902). The tumours tended to be highly malignant, presumably because continued exposure after the initiation of a malignant clone led to its rapid progression, and many of those affected died from the disease. When, in 1936, the German Röntgen Society erected a monument to the memory of those who had died as a result of their work, 169 names were recorded on it (Meyer, 1937) and a further 191 were added in a supplementary list in 1959 (Holthusen *et al.*, 1959 – although Holthusen *et al.* refer only to an additional 190, the names listed add up to 360). These 'radiation martyrs', as they were called (though victims would have been a better term as they did not know beforehand what might happen to them) came from the 22 countries listed in Table I. France tops the list with 65 names, closely followed by Germany with 59 and the US with 55.

Causes of death on the original list were given for all but the 39 US martyrs. Three-quarters were attributed to skin cancer (96/130) and the others to anaemia (18), leukaemia (two), accidents (six) and a variety of causes (eight), ranging from asthenia to chest burns from the transport of radium.

*Leukaemia* Whether leukaemia could be attributed to radiation remained controversial for many years. In 1911 von Jagic *et al.* reported four cases following prolonged exposure and several more were recorded in the next 20 years. Then Aubertin (1931) reported seeing five radiologists with myeloid leukaemia in a period when he had seen the disease in only one non-radiological physician, which led him to conclude that myeloid leukaemia was particularly common in radiologists. A year later, however, Nielsen (1932) could still find records of only 14 acceptable cases in radiation workers in the literature and when, in 1934, Colwell and Russ reviewed the injuries attributable to X-rays and radium they could not decide whether leukaemia should be included. In the same year Marie Curie died of chronic myeloid leukaemia; but it was another 10 years before any epidemiological evidence stronger than Aubertin's (1931) was obtained. Then March (1944) matched the list of members of the American Roentgen Ray Society with the death notices in the *Journal of the American Medical Association* and two radiological journals and found that 4.57% of 173 with known causes were attributed to leukaemia against 0.44% of over 50 000 deaths from known cause among non-radiological physicians recorded in the *Medical Association Journal.* The 10-fold difference was statistically highly significant; but, despite the fact that Furth (1934) had already demon-

*Based on the Hamilton Fairley lecture given to the British Association for Cancer Research on 4 December 1995 and a Röntgen centenary lecture given to the Radiation Research Society on 4 April 1995

Received 28 June 1995; accepted 9 August 1995

**Table I** Numbers of radiation martyrs by country of origin (Meyer, 1937; Holthusen *et al.*, 1959)

| | | | | | |
|---|---|---|---|---|---|
| France | 65 | Russia | 13 | Australia | 2 |
| Germany | 59 | Hungary | 11 | Finland | 2 |
| USA | 55 | Austria | 8 | Israel | 2 |
| UK | 42 | Switzerland | 7 | Portugal | 2 |
| Italy | 29 | Belgium | 5 | Dutch East Indies | 1 |
| Japan | 28 | Denmark | 5 | Greece | 1 |
| Czechoslovakia | 17 | Spain | 4 | Poland | 1 |
| | | | | Yugoslavia | 1 |

strated that irradiation could produce leukaemia in animals, March concluded only that 'If .... a relationship exists between prolonged exposure to radiation and the development of leukaemia, then the fact that a certain percentage of non-radiological physicians are exposed to radiation would tend to increase the validity of the attempted demonstration.'

*Lung Cancer* Other cancers that came to be recognised as caused by occupational exposure were cancers of the lung and bone. The first occurred in the miners of Schneeberg and Joachimsthal in the Harz mountains. The mines had been worked for 500 years and the miners had long been known to have a high mortality from a chest disease, called simply mountain sickness. In 1879, Härting and Hesse showed it was cancer, but they called it a lymphosarcoma. The exceptionally high prevalence of occupational cancer was recorded by Arnstein (1913*a*,*b*), who corrected the histological diagnosis and found that 42% of deaths of miners between 1875 and 1912 were attributable to it. This high rate was confirmed by Rostoski *et al.* (1926) who followed 154 miners and ex-miners for 3¼ years. Twenty-one died and lung cancer was confirmed at necropsy in 13. The mines had been worked successively for silver, nickel, cobalt, bismuth, arsenic and pitchblende before they provided the ore from which, 2 years after Röntgen's discovery, the Curies isolated radium, and they went on to be mined for radium and eventually uranium. The idea that the cancers might be due to radon was proposed in 1921 by Margarete Uhlig, herself a native of Schneeberg, who noted that she had seen the suggestion made 'in an article by a layman'. Rajewsky's (1939) measurements of the radioactivity in the air of the mines averaged about 3 $\mu$Ci ($1 \times 10^5$Bq) $m^{-3}$ and the attribution to ionising radiation was commonly accepted. When, however, Lorenz reviewed the evidence in 1944 he concluded that radon was not the sole cause of the excess. Contributing factors, he thought, might include pneumoconiosis, arsenic and perhaps hereditary susceptibility.

*Bone Sarcoma* The role of radium in the production of bone sarcoma in luminous dial painters was recognised more quickly. Workers in a New Jersey factory had used a phosphorescent zinc sulphide paint, made luminous by the addition of small amounts of radium and mesothorium, and in 1925 Hoffman reported that 12 had persistent infections of the jaw, sometimes with marked anaemia, from one or both of which four had died. Six years later, Martland (1931) reported 18 deaths from occupational disease among 800 young women who had been employed in the factory, all in the small proportion who had worked there for more than 2 years. Most had severe anaemia but in five instances death was due to sarcoma of the bone. Substantial amounts of radium were present in their bones and Martland concluded that the 27% of deaths attributable to sarcoma was so much greater than the 0.1% or less recorded in autopsy series that it provided 'overwhelming evidence that the radioactivity of these dial painters is the true cause of the sarcoma'. Martland's comparison was hardly fair, but the cancers all occurred in bone that showed severe osteitis and the relationship was inescapable.

Martland (1931) noted that McCombs and McCombs (1930) had just advanced the hypothesis 'that cancer is due primarily to mutation in a somatic cell caused, possibly, in some instances by ionization' and he wondered whether 'some such agency is active in the greatly increased prevalence of cancer' in the luminous dial painters. If it was, he recognised that there was unlikely to be a threshold dose below which no effect occurred and he suggested that 'some other types of malignancy may be caused by minute amounts of radioactive substance to which the human body, in its normal environment, is exposed.'

*Other cancers* Other types of cancer were found to occur in tissues that had been scarred as a result of heavy irradiation, including cancers of the lip, larynx, pharynx, thyroid and connective tissue. These occurred, for the most part, 20–40 years after radiotherapy, which had been given for thyrotoxicosis, for tuberculosis affecting the skin, bones and cervical glands, and (less often) for other cancers in neighbouring sites. Proof that they were caused by radiation was lacking; but causation was assumed because they occurred in sites showing radiation scars in which cancer was not normally common. In 1957, Cade could find less than 300 cases of all types of cancer attributed to the use of radiotherapy in the medical literature, including cancers of the skin; but he added 34 that had come under the care of three radiotherapists in London and the actual total must have been much higher.

### *Genetic damage*

The possibility that genetic damage might occur was recognised very early from animal experiments, when Albers-Schönberg (1903) in Hamburg demonstrated that X-rays could damage rabbits' testes. It was not, however, until 1907 that transmissible genetic damage was reported, when Bardeen, an anatomy professor at the University of Wisconsin, produced congenital defects in toads by irradiation of the parents' spermatazoa. Twenty years later, Muller (1927), a zoologist at the University of Texas, showed that X-rays could produce mutations in fruit flies and that the prevalence of the effect was approximately linearly proportional to the dose. No observation had been made on humans; but the evidence for the production of hereditary mutations was held to be of universal application and its applicability to humans was not questioned.

### *Criteria for protection*

The recognition of the acute effects had led rapidly to the introduction of techniques to reduce the direct exposure of the operator and to limit the dispersal of the rays, once it was recognised that the rays themselves were responsible. The discovery of the possibility of damage to the testes heightened concern and by 1905 the German Röntgen ray expert who discovered the damage was 'encased from Schnurrbart to foot in a veritable suit of armour' (Butcher, 1905). It was not, however, until the First World War, when many new operators had to be recruited to provide the rapid expansion of X-ray facilities required to deal with war injuries, that the need was felt for codes of practice, as was recommended by the Röntgen Society in Britain in 1915.

Little attention seems to have been paid to such informal advice and it was superseded by the formal recommendations of protection committees set up by the American Roentgen Ray Society (1922) in 1920 and a group of concerned British scientists in 1921. The recommendations of the self-styled British X-ray and Radium Protection Committee (1921) were published first and were subsequently accepted with minor modifications for international use by the second Congress of Radiology in Stockholm in 1928 (International X-ray and Radium Protection Committee, 1928). With the introduction of a measure of dose in the same year, the recommended limit of exposure was set at 1 rad per week in 1934, where it remained until after the Second World War.

### State of knowledge in mid-century

From 1928, until the explosion of the atomic bombs over Hiroshima and Nagasaki, 3 months short of the 50th anniversary of Röntgen's discovery, it was thought that clinical experience aided by animal experimentation had found a way to use ionising radiations safely, without causing an occupational hazard for the operator or long-term side-effects for the patient. The acute effects and the risk of progressive anaemia had been avoided or, in the case of radiotherapy, kept within acceptable limits; the risk of causing cancer, which had not been accepted as caused by mutations, was generally thought to have been avoided by avoiding macroscopic damage to tissues (Glücksmann, 1952); and the genetic effect, which was accepted as due to mutations and capable of being caused by any dose of ionising radiations however small, could be kept to negligible levels by protecting the gonads. Only March's (1944) epidemiological observation, made a year before the explosions, gave cause to think that any long-term effects might still be being produced.

### The impact of nuclear power

### *Establishment of the Atomic Bomb Casualty Commission*

Following the atomic explosions, there was great public concern about the possible effects that the radiation might have had. By far the greatest proportion of the approximately 180 000 deaths was the direct result of blast and heat. Several thousand of the immediate survivors, however, died shortly afterwards as a result of acute radiation sickness and thousands more experienced acute symptoms and recovered. What might happen later to those who recovered was unclear. It was recognised that knowledge of the long-term effects of substantial amounts of whole-body irradiation was incomplete and the joint commission of the US Army and Navy, which visited Japan shortly after the war, recommended a long-term study of the survivors to determine any possible medical and biological effects. The proposal to set up an Atomic Bomb Casualty Commission (ABCC) in conjunction with the Japanese National Institute of Health was approved by Truman in November 1946 and an ABCC office was opened in January 1948 with long-term financial support from the US Atomic Energy Commission. Large programmes of research were initiated into the genetic and somatic effects of radiation, as seen in the survivors of the two explosions, and a new era was opened (Cannan, 1962).

The genetic programme under the direction of Neel and Schull was epidemiological in character from the start, as it set out to determine the prevalence of congenital anomalies, the sex ratio at birth, stillbirth and neonatal death rates, birthweights and anthropometric measurements in infancy for all children born in the two cities. Data were collected by research workers shortly after the births had occurred and a supplementary questionnaire was completed by, and a blood sample taken from, all mothers whose pregnancy terminated abnormally and a 'random' 10% of all other mothers. Autopsies were obtained for about half of all stillbirths and neonatal deaths and a quarter of surviving infants were re-examined at a central clinic at 9 months of age. Parents were classified in five exposure groups as shown in Table II and compared with regard to age, parity, economic status, consanguinity, prevalence of syphilis, previous induced abortion and degree of cooperation. The programme began in stages from 1948 and was closed in January 1954 because of the rapid reduction in the birth rate, when the only suggestive evidence of variation in the direction expected from genetic theory was the trend in the sex ratio (Neel *et al.*, 1953; Neel and Schull, 1956).

The somatic effects programme, by contrast, developed piecemeal. It started by conducting surveys and, in the classical clinical way, by selecting a relatively small group of exposed survivors and arranging for them to have regular clinical examinations; the so-called Adult Medical Survey. Survivors were identified in a radiation census in 1949 and a sample census carried out by the ABCC a year later but there was, at that time, no prospect of obtaining estimates of dose for individuals. Exposed survivors were, therefore, classified according to their distance from the hypocentre at the time of the explosion and the presence or absence of acute symptoms attributable to irradiation. The surveys quickly provided conclusive evidence that irradiation increased the risk of leukaemia (Folley *et al.*, 1952), cataracts (Cogan *et al.*, 1952) and mental retardation in children most proximally exposed *in utero* (Plummer, 1952), but the plan for repeated clinical examinations proved to be ill-conceived. By 1954, it was seeking to examine regularly some 5000 people, who had been exposed within 2000 m and had suffered symptoms attributable to irradiation, and a control group of men and women matched for sex and age, who had been exposed beyond 2500 m, and it was foundering in the face of negative findings and declining participation. There was, consequently, thought of closing it down (Beebe, 1979).

### *Reaction to the hydrogen bomb test*

Just then another event occurred that altered the perspective of governments and their scientific advisers throughout the world. In June 1954, a hydrogen bomb was exploded over Eniwetok in the Pacific, which had 1000 times the power of the Hiroshima and Nagasaki bombs, and radioactive fallout

**Table II** Classification of parents by exposure (after Neel and Schull, 1956)

| *Distance from hypocentre* | *Shielding* | *Symptoms*[a] | *Group* |
|---|---|---|---|
| Unexposed | | | 1 |
| Over 3000 | | + or – | |
| 2000–2900 | Some | – | 2 |
| 1500–1900 | Light or moderate | – | |
| Under 1500 | Heavy | | |
| 2000–2900 | None | – | |
| 1500–1900 | Light | – | 3 |
| 1000–1400 | Light or moderate | – | |
| Under 1000 | Moderate | – | |
| 1000–1900 | None | – | 4[b] |
| Under 1000 | Light or none | – | |
| Under 3000 | None or some | + | 5[b] |

[a]Epilation, petechiae, gingivitis. [b]Categories 4 and 5 are usually combined.

was distributed over the whole globe. Further test explosions seemed certain to be carried out by competing powers and determination of the quantitative effects of small doses of radiation became a burning issue. National committees were appointed in the UK and the US to review the evidence. Their reports made it clear that no quantitative estimate of the risks could then be made (Medical Research Council, 1956; National Academy of Sciences, 1956*a*) and an immense amount of research was initiated. Epidemiology by this time had been shown to be capable of contributing to knowledge of the aetiology of non-infectious disease and radio-epidemiology moved from the wings to the centre of the stage.

Keith Cannan had by then become Chairman of the Division of Medical Sciences at the National Academy of Sciences and the ABCC was one of his responsibilities. He quickly realised that the existing programmes were not going to provide the information needed and he sought advice from an *ad hoc* committee under the chairmanship of Thomas Francis Jr. A unified plan was recommended to determine the cause specific mortality of a cohort of some 100 000 persons (subsequently increased to about 120 000) who were selected from nearly three times that number resident in Hiroshima or Nagasaki at the time of the national census on 1 October 1950 and whose history of exposure was known (Francis *et al.*, 1955). This was immediately accepted and the sample, with some minor modifications (Jablon *et al.*, 1965; Beebe *et al.*, 1971) became the basis for the Life Span Study, which is still continuing and has provided the principal evidence on which our current knowledge of the long-term effects of radiation is based. To it was added a mortality study of 2800 individuals exposed *in utero* and non-exposed controls (Kato, 1971) and the registration of all cases of cancer, irrespective of the individual's subsequent survival (Beebe and Hamilton, 1975) and research was begun to enable tissue doses to be estimated for each member of the cohort.

In the UK, a smaller study was instigated by the Medical Research Council, with the specific purpose of determining the quantitative relationship between X-ray dose and the incidence of leukaemia (Medical Research Council, 1956; Court Brown and Doll, 1957). Data were obtained for 14 000 patients who had been given radiotherapy for ankylosing spondylitis and 1500 patients treated by other means and this study has also been continued to the present day and expanded to include mortality from all types of cancer and a detailed assessment of the doses received in each major organ (Weiss *et al.*, 1994).

### The contribution of modern radioepidemiology

In the years that followed, many other epidemiological studies were carried out and more detailed information about the qualitative and quantitative effects of ionising radiation obtained than about any other noxious agent except, perhaps, cigarette smoke, The literature has been reviewed regularly by the United Nations Scientific Committee on the Effects of Atomic Radiation (UNSCEAR) from 1958 to 1994 and the International Commission on Radiological Protection (most recently in 1990) and periodically by the US National Research Council (most recently in 1988 and 1990) and other similar bodies and it would be impossible to do justice to it here. I shall, therefore, just summarise the risks as I see them now and draw attention to the limits of their reliability.

Four effects have been postulated from doses less than those required to produce acute effects or macroscopic damage to tissues, either on the basis of animal experiments or from human experience: namely, the production of mutations in germ cells leading to the development of congenital anomalies or hereditary disease in offspring, congenital defects from irradiation *in utero*, an increased rate of non-specific ageing and an increased risk of cancer.

### *Genetic effects*

That mutations would have been produced in the sperm and ova of the survivors of the explosions was never doubted. The risk was assumed to be proportional to the dose received, when the dose was small enough not to produce infertility, and damage to the world's genetic stock was long thought to be the principal hazard of the fallout from the atmospheric testing of nuclear weapons. In 1956, however, the British Medical Research Council, in its report on the hazards of nuclear radiation, could base its estimates of the dose required to double the mutation rate in humans only on the assumption that it was similar to that of the animals and plants for which it had reliable information. It concluded that the best estimate of the doubling dose was between 30 and 80 rads (0.3–0.8 Gy). Even the lowest estimate of 3 rads (0.03 Gy) based on the assumption that all mutations were normally caused by natural radiation, was not thought to be contradicted conclusively by what had by then been found in Japan.

More precise determination of the susceptibility of human gonads to irradiation was, however, so important that the ABCC initiated a fresh programme to obtain further data on the trend in the sex ratio at birth with parental dose and to compare the lifespan of children, one of whose parents had been irradiated, with that of suitable controls. The first objective was attained by the end of 1962, by which time the sex was known for over 140 000 children, 74 000 of whom had one or more exposed parents. The regression of the sex ratio on dose was then found to be trivial and in the opposite direction to that postulated (Schull *et al.*, 1966) and no further study of this effect seemed worthwhile.

The second objective was, in contrast, expanded to include comparisons of cancer incidence, cytogenetic changes and mutations altering the electrophoretic mobility or activity of 30 proteins and the initial findings have been reanalysed in the light of the estimates of dose obtained in 1986. The results of 40 years' observations were summarised by Neel and Schull and their colleagues in 1990. No statistically significant effects were seen with any of eight indicators. For six of the indicators, the regressions on dose are summarised in Table III. For three of them, the direction of the trend is contrary to what would be expected from damage to parental germ cells. The two other indicators were not being summarised in the same way. One, balanced structural rearrangement of chromosomes, showed a non-significant increase in the prevalence of aberrations with increasing dose, but a lower prevalence in children of all exposed parents (0.22%) than in children of controls (0.31%). No regression on parental dose was calculated, because it has not yet been possible to separate with certainty the abnormalities inherited from the parents from those that had arisen *de novo* in the germ line. The other, the growth and development of the offspring, is not amenable to similar quantitative analysis. So far, measurements into school ages have not revealed any differences that could be related to parental exposure.

*Estimation of doubling dose* Not all these indicators are likely to have an equal contribution from spontaneous muta-

**Table III** Regression on parental dose of six indicators of genetic damage (after Neel *et al.*, 1990)

| *Indicator* | *Regression on dose (Sv) ± standard error* |
|---|---|
| Untoward pregnancy outcome | 0.00264 ± 0.00277 |
| Mortality from non-malignant disease | 0.00076 ± 0.00154 |
| Incidence of cancer under 20 years | – 0.00008 ± 0.00028 |
| (most genetic subset) | (– 0.00005 ± 0.00013) |
| Sex chromosome aneuploidy | 0.00044 ± 0.0009 |
| Mutation altering serum or blood protein | – 0.00001 ± 0.00001 |
| Offspring maleness (mothers only) | 0.0027[a] ± 0.0040 |

[a]Genetic theory predicts a decrease in ratio.

tion and Neel *et al.* (1990) calculate that the most probable gametic doubling dose of acute low LET radiation is between 1.7 and 2.2 Gy. For low dose rate exposures, mouse experiments suggest that the effect should be halved, leading to a doubling dose of the order of 4 Gy or somewhat more — 5 times the upper limit of the Medical Research Council's estimate in 1956. This estimate does not take into account the few observations of reciprocal translocations (which were equal in the two populations) nor the sex ratio results (which were counter to the genetic hypothesis) nor the data on growth and development (which revealed no hint of a radiation effect). If these other indicators could also be taken into account, the estimate of the doubling dose would be increased further.

*Paternal radiation and childhood leukaemia* The only material challenge to this conclusion came from Gardner *et al.* (1990) when they adduced evidence to suggest that paternal irradiation in the course of employment at the nuclear reprocessing plant in Sellafield was responsible for the small cluster of young people with leukaemia that occurred in the village of Seascale, 3 km south of the plant. Seven cases were seen between 1955 and 1983, which was about ten times the number expected. A case-control study of children born and resident in the county found that the risk was concentrated in children whose fathers had received substantial doses of ionising radiation at work before the child's conception (Table IV). Support for Gardner's idea came from studies in which radiation histories (mostly of medical X-rays) had been given by parents some years after the event and were, therefore, liable to recall bias. Studies in which exposures had been recorded objectively, as in Yoshimoto *et al.'s* (1990) study of the children of Hiroshima and Nagasaki survivors and in studies in Canada (McLaughlin *et al.*, 1993), Scotland (Kinlen *et al.*, 1993) and the south of England (Roman *et al.*, 1993) have not shown any similar effect, nor has there been any excess of childhood leukaemia in other parts of the county in which Sellafield is situated, despite the fact that 92% of the births to Sellafield employees occurred outside Seascale and the fathers had received 93% of the total preconception dose (Parker *et al.*, 1993). Childhood leukaemia, moreover, seems seldom, if ever, to be a dominantly inherited disease (Hawkins *et al.*, 1995) and the size of the effect postulated by Gardner *et al.* (1990) is biologically implausible for such relatively small doses. Review of the totality of the evidence leads to the conclusion that the cluster was not due to chance, but that the association with paternal irradiation largely or wholly was (Doll *et al.*, 1994).

*Future prospect* Barring accidents, it seems unlikely that any better estimate of the genetic risk is likely to be obtained from any other human population and further precision can be expected only from the genetics programme now being undertaken by the Radiation Effects Research Foundation in Hiroshima. This is intended to establish 1000 or so constellations of lymphocytoid cell lines from fathers, mothers, and children, half from exposed families and half from controls, which can be examined for mutation of selected proteins.

### *Congenital defects from irradiation* in utero

*Serious mental retardation* The second effect, damage of the developing embryo or fetus, has caused radiologists much anxiety. That exposure of the abdomen of pregnant women to doses or the order of 200–500 mGy might cause the infant to be born with a small head and to be mentally retarded had long been suspected clinically, but was established by observations on the survivors of the atomic bomb explosions.

A total of 1544 children born to women exposed during pregnancy have been followed and 30 have been classed as severely mentally retarded on clinical grounds. The prevalence in relation to dose and post-ovulatory age is shown in Table V, after exclusion of three children with Down syndrome. The maximal effect was seen when exposure occurred during the 8th–15th week of pregnancy, when cortical neurones are most actively produced. At this period some effect was seen with doses down to 50 mGy, but lack of

**Table IV** Relative risk of leukaemia in young persons by father's occupational exposure to ionizing radiation (after Gardner *et al.*, 1990)

| *Father's dose before child's conception* | *No. of cases* Leukaemic children | Area controls | *Relative risk* | *95% Confidence limits* |
|---|---|---|---|---|
| Total | | | | |
| 0 | 38 | 253 | 1.00 | – |
| 1–49 mSv | 3 | 19 | 1.12 | 0.31–4.05 |
| 50–99 mSv | 1 | 11 | 0.69 | 0.08–5.73 |
| ⩾ 100 mSv | 4 | 5 | 6.24 | 1.51–25.76 |
| Previous 6 months | | | | |
| 0 | 38 | 262 | 1.0 | – |
| 1–4 mSv | 3 | 18 | 1.30 | 0.32–5.34 |
| 5–9 mSv | 1 | 3 | 3.54 | 0.32–38.88 |
| ⩾ 10 mSv | 4 | 5 | 7.17 | 1.69–30.44 |
| No dose | 38 | 253 | 1.00 | – |
| Any dose | 8 | 35 | 1.71 | 0.68–4.26 |

**Table V** Prevalence of severe mental retardation in individuals exposed *in utero*[a] (after UNSCEAR, 1993)

| *Post-ovulatory age (weeks)* | *Dose (Gy)* <0.01 (0)[b] | 0.01–0.09 (0.05) | 0.10–0.49 (0.23) | 0.50–0.99 (0.64) | ⩾ 1.0 (1.38) |
|---|---|---|---|---|---|
| 8–15 | 2/255 (0.8%) | 2/44 (4.5%) | 1/57 (1.8%) | 3/15 (20.0%) | 9/12 (75.0%) |
| 16–25 | 2/308 (0.6%) | 1/55 (0.8%) | 0/57 (0.0%) | 0/16 (0.0%) | 3/8 (37.5%) |
| 0–7 or ⩾26 | 4/504 (0.8%) | 0/102 (0.0%) | 0/92 (0.0%) | 0/10 (0.0%) | 0/6 (0.0%) |

[a]Three individuals with Down syndrome excluded (doses 0 at 36 weeks, 0.29 Gy at 13 weeks and 0.56 Gy at 12 weeks). [b]Mean uterine absorbed dose.

knowledge of the mechanism by which mental retardation is produced makes it difficult to know *a priori* what type of dose–response relationship should be expected (UNSCEAR, 1993). From the 16th–25th week of pregnancy an effect was seen only with doses greater than 1 Gy; at other periods no effect was seen.

*Intelligence test scores* Intelligence test scores of children not mentally retarded show a reduction which is similarly limited to those exposed in the 8th–25th weeks of pregnancy and is most marked in the 8th–15th week. The results are shown in Figure 1. Qualitatively, the findings are consistent with the interpretation provided by UNSCEAR (1993) that there is a dose-related shift in IQ that is large enough to explain the increase in the number of children clinically classified as mentally retarded.

*Small head and seizures* The only other effects to have been observed in liveborn infants are a reduction in head size and seizures. The latest analyses, which allow for normal variation, provide no reason to think that small heads were caused after the 15th week of pregnancy. Before that period 12 were associated with mental retardation and 18 were associated with a generalised impairment of growth, which, unlike mental retardation, also occurred with exposure in weeks 1–7 (Otake and Schull, 1993).

Seizures at 2 years of age or over, were related to dose only when the child had been exposed *in utero* at a post ovulatory age of 8–15 weeks. After exclusion of those associated with severe mental retardation and those attributable to some extraneous cause, seizures (which occurred in about 1% of unexposed children) were also related to dose in an apparently linear manner, without any statistical reason to postulate a threshold (Dunn *et al.*, 1990).

*Total effects on development of brain* The quantitative risk of all these effects on the development of the brain, which are plausibly related to dose in a linear way when exposure occurs at 8–15 weeks, are summarised in Table VI. At 16–25 weeks the risks are much less and cannot be related to dose at levels less than 0.5 Gy. Uncertainty about the biological mechanisms and the absence of appropriate human data limit these findings to the effects of acute doses and prohibit their extrapolation to doses given at low dose rates.

*Abortion* No other risk of congenital defect has been seen, possibly because any dose large enough to produce such an effect in the first 7 weeks also causes the embryo to be aborted, as about half the embryos that were exposed to 0.5 Gy or more in this period were lost (Committee for the Compilation of Material on Damage Caused by the Atomic Bombs in Hiroshima and Nagasaki, 1981).

*Non-specific ageing*

The third effect, an increased rate of non-specific ageing, was predicted on the basis of animal experiments in which irradiated rodents experienced a shortening of life proportional to dose with a 7% loss per $LD_{50}$ (National Academy of Sciences, 1956*b*). Within weeks, the idea spread round the world that 1 rad led to the loss of expectation of about 10 days' life (4.7% shortening of life per 100 rad, Storer and Sanders, 1958) and this, or something very similar, was supported when Warren (1956) reported that US radiologists experienced a loss of 5.2 years of life, or 11% of the adult life span, in comparison with physicians with no known contact with radiation. Both claims were, in fact, unjustified, being based on a misuse of data for the average age at death. The rodents suffered an increased age-specific mortality from cancer that reduced their lifespan and consequently the age at death from other causes, while Warren had not taken account of the peculiar age distribution of radiologists that allowed them to have the same mean age and a lower mean age at death without having any higher mortality at any given age. Subsequently, however, Seltser and Sartwell (1965)

**Figure 1** Mean IQ scores recorded in individuals exposed *in utero* to the atomic explosions at Hiroshima and Nagasaki by post-ovulatory age (United Nations Scientific Committee on the Effects of Atomic Radiation, 1994; Schull *et al.*, 1988). ●, >0.01 Gy; ○, 0.01–0.09 Gy; ■, 0.1–0.49 Gy; □, >0.5 Gy; ▲, 0.5–0.99 Gy; △, >1.0 Gy.

**Table VI** Quantitative risks of damage to fetal brain from irradiation at post-ovulatory age 8–15 weeks (after UNSCEAR, 1993)

| *Damage* | *Risk[a] from Exposure to 1 Gy* |
|---|---|
| Serious mental retardation | Increased from 0.8% to 40% |
| IQ | Loss of 25–30 per cent points |
| School performance | 50th percentile reduced to 10th percentile |
| Unprovoked seizure in absence of serious mental retardation | Increased 25 times at ages 2 years and over |

[a]Risk linearly proportional to dose.

showed that the mortality of US radiologists was higher than that of non-radiological physicians and higher still than that of US ophthalmologists and ear-nose-throat specialists, both from cancer and from other causes, and they concluded that the results 'warrant the inference that occupational exposure to ionizing radiation on the part of physicians has in the past produced a non-specific life shortening effect'. British radiologists, in contrast, were found to have a lower mortality from causes other than cancer than other doctors and other men in the same socioeconomic class, even if they began practice before 1921 when stringent precautions against exposure began to be taken (Smith and Doll, 1981). No general life-shortening effect has been observed in the major studies of radiation workers whose doses of radiation have been measured (Cardis *et al.*, 1995), nor in the Life Span Study at doses less than 2 Gy (Shimizu *et al.*, 1991). The weight of the evidence, in my opinion, suggests that the idea should be abandoned, unless some new experimental evidence is obtained to revive it.

*Risk of cancer*

The fourth effect, an increased risk of cancer, came gradually to be accepted as due to one or more somatic mutations, partly as a result of epidemiological observations in the mid 1950s (Court Brown and Doll, 1957; Lewis 1957) but even as late as 1960 it continued to be resisted by some distinguished radiobiologists (Brues, 1960). With acceptance of its mutational origin, the risk of cancer displaced genetic effects as the principal cause of public concern and the dose tolerated for radiation workers and the general public has been progressively reduced. The principal quantitative evidence has come from the Life Span Study, but it has been extended, modified, and checked by the results of biological experiments and epidemiological studies of patients irradiated for medical reasons, people exposed at work, children exposed to fallout from the testing of nuclear weapons and populations exposed to different amounts of natural radiation by virtue of their place of residence.

*Radon in houses* For a few risks the Life Span Study is of no help, as it provides no information about the effects of high linear energy transfer radiation or of any radionuclides absorbed internally, the most important of which is the effect of radon in house air.

That radon causes a risk of lung cancer roughly proportional to dose and no material risk of any other type of cancer is abundantly clear from 11 major studies of men exposed to radon in mines (Lubin *et al.*, 1995; Darby *et al.*, 1995). Extrapolation from the experience of miners is, however, fraught with difficulty, for the risk varies not only with total exposure and dose rate but also, in a way specific for lung cancer, with time since exposure occurred and in a complex way with the amount smoked. If this were not enough, there are the additional difficulties of extrapolating from male miners who were exposed in adult life, to members of the general public, who breathe less deeply, breathe air that is less contaminated with dust, were exposed in childhood and include women. Temporary solutions have been proposed by the US National Research Council (1991) and Lubin *et al.* (1995) and the latter have used them to estimate that the proportions of lung cancer deaths attributed to domestic radon in the US are 10% in men and 12% in women, less in smokers and rising to 28% and 31% respectively in non-smokers. Lubin *et al.'s* (1995) formulae have not yet been applied in Britain, but the proportions would be likely to be about half those in the US, corresponding to the lower mean concentration of the gas (arithmetic mean 20 Bq $m^{-3}$ against 49 Bq $m^{-3}$).

In these circumstances estimates of the effect of radon in houses, which may vary 100-fold, need to be validated by direct observations on people with and without lung cancer, with known smoking habits and measured concentrations of radon in their houses. Such studies, however, also have their difficulties, including the need to measure concentrations in past places of residence, which may have been pulled down or altered, random errors in measurement and differences in behaviour that affect the dose that individuals receive in a given environment. So far seven such studies have been reported, four of which have led to estimates of risk that are not very different from those extrapolated from the experience of miners (Schoenberg *et al.*, 1990; Ruosteenoja, 1991; Pershagen *et al.*, 1992), while three have not suggested any risk at all (Blot *et al.*, 1990; Alavanja *et al.*, 1994; Letourneau *et al.*, 1994). Several others are in train and the best estimate of risk should eventually be obtained from the pooled results of the most reliable studies. If then we add on the complexities of the model required to determine the dose received by the cells capable of giving rise to lung (or rather bronchial) cancer, it follows that these epidemiological observations can as yet tell us little or nothing about the quality factor appropriate for alpha radiation. Nor can the much fewer observations of the effect of the intake of radium on the risk of bone sarcoma or of thorium on the risk of liver cancer and leukaemia (National Research Council, 1988).

*Irradiation of fetus* For another risk the evidence from the Hiroshima and Nagasaki survivors and case–control studies of affected patients is conflicting: namely, the risk of childhood cancer from irradiation *in utero*. Two children developed cancer under 15 years of age out of 1263 exposed to the bombs prenatally with less than one expected (0.73) and the estimated excess per Gy was 0.5% with 95% confidence limits of −0.2% to 2.4%. Neither child had leukaemia and the upper 95% confidence limit for this disease was 1.1% per Gy (Yoshimoto *et al.*, 1988). Both these upper limits are substantially less than the excesses expected to occur from the results of the case–control studies, as estimated by the National Radiological Protection Board (Muirhead *et al.*, 1993). The results of the case–control studies are, however, so consistent, based on such large numbers, and shown not to be plausibly explained by recall bias or confounding (Hewitt *et al.*, 1966; Bithell and Stewart, 1975; Monson and MacMahon, 1984; Mole, 1990) that they cannot easily be dismissed. A causal explanation for the observed relationship is, moreover, supported by the evidence from the Oxford Survey of Childhood Cancers, which shows that the risk increased with the number of films to which the fetus was exposed in the last trimester of intra-uterine life and declined compatibly with the temporal decline in obstetric dose (Bithell, 1989). On detailed examination the various objections that have been made to a causal explanation seem likely to be invalid (Doll and Wakeford, 1995) and the relatively small excess observed in children exposed prenatally in Hiroshima and Nagasaki is best attributed to an unusual effect of chance. On this basis, the best estimate of the risk of childhood cancer from irradiation *in utero* is 6% per Gy and of childhood leukaemia 2.5% per Gy (Muirhead *et al.*, 1993), the last of which is only slightly greater than that from exposure in early childhood (1.8% per Gy).

*External irradiation after birth* For most ordinary exposures, the results of the Life Span Study are pre-eminent. Nearly 7000 deaths from cancer and over 8500 registered cases have been observed in a population of some 76 000 people with individually estimated doses, and the results have led to the conclusion that the lifetime risk from fatal cancer is about 10% per Sv with about a tenth of the deaths attributable to leukaemia, that the risk is linearly proportional to dose between about 0.2 and 4 Sv, and that linear extrapolation to lower doses goes through or very close to zero. On the basis of animal experiments it has usually been assumed that extrapolation to the effects of doses below 0.2 Sv, and certainly to the effect of such doses given at low dose rates, requires division by two (International Commission on Radiological Protection, 1990). It is now clear, however, that the analyses that led to these conclusions were too simple.

Physically, it appears from measurements of specific radionuclides in concrete and soil that the neutron doses at distances greater than 1 km from the hypocentre of the explosion over Hiroshima have been underestimated and this may lead to a reduction in dose estimates by 10–20% (Straume *et al.*, 1992; UNSCEAR, 1994). Clinically, a review of autopsy records suggests that the mortality attributed to cancer has been underestimated, particularly in the older age groups. While not affecting estimates of relative risk, this suggests that absolute excess estimates need to be increased by about 10% (Sposto *et al.*, 1992) which largely cancels out the error due to the underestimation of neutron dose.

Statistically, random errors in dose estimates lead to a downward bias in the estimated risk per unit dose and may also distort the shape of the dose–response curve, so as to make an upward curvature approximate linearity. Different methods have been used to estimate the effect of such errors. A conservative estimate by Pierce *et al.* (1990) implies that the risk estimates are too low: for solid cancers by 7–11%, and for leukaemia by 4–7%. Statistical analysis, taking these errors into account, shows that the dose–response relationship for leukaemia is significantly better fitted by a linear quadratic relationship than by a linear one and that the best estimate of the factor by which the slope of a linear model should be divided to give the slope at low doses is 2.2. There is, however, no compelling statistical reason to abandon a linear relationship for fatal cancers other than leukaemia, as the best estimate of the comparable reducing factor is 1.3, which is not significantly greater than 1 (Pierce and Vaeth, 1989, 1991).

It is the biological considerations, however, that are likely to be the most important: in particular the possibility of variation in susceptibility by sex, age, organ irradiated and background risk. Variation by age is particularly important as the excess relative risk has proved to be higher for exposure in childhood and youth than at older ages and determination of the attributable lifetime risk for cancers

**Table VII** Estimate of lifetime risk of death from cancer other than leukaemia following acute whole body exposure to radiation [a](after UNSCEAR, 1994)

| *Assumption for future relative risk* | *Risk of exposure induced death (%) Following 0.2 Sv* | *Following 1 Sv* |
|---|---|---|
| Constant | 2.4 | 10.9 |
| Decline to excess risk at age 50 years | 1.9 | 9.2 |
| Decline to zero excess risk at age 90 years | 1.6 | 7.5 |

[a]Population with age distribution of Japan in 1985.

other than leukaemia depends crucially on whether the excess relative risk remains constant throughout life for those exposed in youth, as it has done approximately for those exposed at older ages or whether it falls, as the cancer incidence data suggest (Thompson *et al.*, 1994). The importance of this is indicated in Table VII, which shows the variation in the estimated lifetime risks of exposure-induced death from cancers other than leukaemia from doses of 0.2 and 1.0 Sv depending on the trend in risk in young people after 1987.

How far there is any variation in the susceptibility of organs is unclear, apart from the greater susceptibility of the marrow to the induction of myeloid and acute lymphatic leukaemia and the reduced (even zero) susceptibility to chronic lymphatic leukaemia. The excess relative risk per Sv and its 90% confidence limits for 18 types of cancer, as observed in the Life Span Study over the period 1958–87, is shown in Figure 2. Nearly all the confidence limits overlap. The high relative risk of breast cancer is not seen in irradiated cohorts in western countries and may result from the low normal risk of breast cancer in Japan. There appears, however, to be a generally low relative risk for cervix cancer, Hodgkin's disease, and possibly non-Hodgkin's lymphoma. There is evidence too from several studies to suggest that the temporal distribution of the risk varies by site, the risk of lung cancer, for example, falling 20 years after exposure and the risk of bladder cancer starting late and persisting longer. It may consequently prove necessary to work out dose–response relationships separately for each of the main types of cancer.

**Figure 2** Excess relative risk per Sv and 90% confidence limits for 18 types of cancer observed in the Life Span Study of survivors of the Hiroshima and Nagasaki explosions (1958–87) (United Nations Scientific Committee on the Effects of Atomic Radiation, 1994; Thompson *et al.*, 1994).

The most important consideration may, however, prove to be the variation in risk with the size of the background risk, which may subsume much of the variation with sex. For it is variation with background risk, or lack of it, that determines the possibility of generalising observed risks from one population to another. Hitherto it has been assumed that the excess relative risk will be the same for all populations. The current method may prove to be right for some cancers, but for others it is almost certainly wrong. For lung cancer, for example, the excess relative risk is much greater for females than for males, and this may indicate that the interaction with smoking is nearer additive than multiplicative, as has been found in the studies of men exposed to radon in mines and from *in vitro* studies of cell transformation (Piao *et al.*, 1990). If similar variation holds for other types of cancer, it will vitiate the generalisation of site-specific risks.

Important though these qualifications are for estimating the precise effect of a given dose in different situations, none of them will, I think, modify our general conclusions about the nature of the dose–response relationship. Nor, taken all together, should they alter our estimate of the regression of risk on dose by as much as 2-fold in either direction.

*Effect of very small doses* What then can we conclude about the risks extrapolated down to the low doses of modern radiography and nuclear medicine and the minute doses to which we are continuously exposed from natural sources in the environment and our own bodies? Epidemiology can take us so far, but sooner or later we have to resort to a model for quantification. Given a model, which it has been shown fits human experience at moderate doses, epidemiology can at least check that the risk extrapolated down to very low doses has not been seriously underestimated. A model that results in a linear, or a linear quadratic, relationship with dose without a threshold accords well with the effects observed for doses of 100 mGy and above up to about 4 Gy, when killing effects begin to distort the curve. In my own view, which may not be widely shared, the observations on the irradiated fetus justify extending the model down to about 10 mGy, but whether there is such an efficient repair mechanism for damage to DNA or a reaction to stress that greatly reduces the effect of tiny doses is something that observations on humans are unable to decide.

So far as leukaemia is concerned, the evidence of the effect of the accumulation of the tiny doses received in Utah from the testing of nuclear weapons in Nevada (Stevens *et al.*, 1990) and of those received in the Nordic countries from the testing of nuclear weapons worldwide (Darby *et al.*, 1992) shows that the current model does not seriously underestimate the effect and may even be thought to give it some support. And if this is true of leukaemia it is likely also to be true for other cancers caused by irradiation. If then we allow that doses received at very low dose rates have only half the effect that the dose–response relationship at high dose rates would suggest, we can conclude that natural radiation in the UK, other than radon, is responsible for about 1.4% of all fatal cancers and that the effect of medical uses of ionising radiation can be deduced *pro rata*, depending on the age of the subject and his or her expectation of life.

### Conclusion

In this review I have shown that clinical intuition served the early exponents of Röntgen's discovery reasonably well. It enabled them to detect the acute effects quickly, to recognise the potential of X-rays to cause cancer in many tissues and to control exposure so as to avoid aplasia of the marrow. It failed to achieve consensus about the risk of leukaemia and completely failed to recognise that the risk of cancer was proportional to cumulative dose and that doses less than those that caused gross mental retardation could damage the fetal brain. Not surprisingly it failed to develop any sense of the extent of the genetic hazard.

The development of epidemiology since the mid-century has provided quantitative estimates of all these risks with greater precision than is available for most other stochastic effects. There remain some uncertainties, many of which are capable of being cleared up within a few years. Precision in the estimation of very small risks by epidemiological methods is, however, a will-o'-the-wisp. Sooner or later we have to resort to a model for quantification, which observations on humans can help to define. Given a model, investigation of the effect of very small doses can then ensure that the risk extrapolated from higher doses is not substantially underestimated, and this I believe it has already done.

**Acknowledgements**

I am most grateful to many colleagues who have helped me find my way through the old literature, particularly to Mrs Roedler-Vogelsang of Munich for details of the early radiation martyrs and some early references, to G Beebe and R Miller for information about the early years of the ABCC, to S Darby and R Wakeford for help in assessing the current position, and to the United Nations Scientific Committee on the Effects of Atomic Radiation and the investigators for permission to reproduce Figures 1 and 2.

**References**

ALAVANJA MCR, BROWNSON RC, LUBIN JH, BROWN C, BERGER C AND BOICE JD. (1994). Residential radon exposure and lung cancer among non-smoking women. *J. Natl Cancer Inst.*, **86**, 1829–1837.

ALBERS-SCHÖNBERG HE. (1903). Ueber eine bisher unbekansite wirkung der Röntgenstrahlen auf der organismus. *Münchener medizin Wochenschr*, **1**, 1859.

AMERICAN ROENTGEN RAY SOCIETY. (1922). Report of Roentgen Ray Protection Committee.

ARNSTEIN A. (1913*a*). Über den sogenannten 'Schneebergen Lungenkrebs'. *Verhandl Deutsch Path Gesellschaft*, **16**, 332–342.

ARNSTEIN A. (1913*b*). Sozialhygienische Untersuchungen über die Bergleute in den Schneeberger Kobaltgruben, insbesondere über der Vorkommen des sogennanten Schneebergen lungenkrebses. *Österr San-Wes, Wien*, **25**, 64–83.

AUBERTIN C. (1931). Leukaemia in radiologists. *Gaz méd de France*, pp. 333–335.

BARDEEN CR. (1907). Abnormal development of toad ova fertilized by spermatozoa exposed to Roentgen rays. *J. Exp. Zool.*, **4**, 1–44.

BEEBE GW. (1979). Reflections on the work of the Atomic Bomb Casualty Commission in Japan. *Epidemiologic Rev.*, **1**, 184–208.

BEEBE GW AND HAMILTON HB. (1975). Future research on atomic bomb survivors. *J. Radiat. Res.*, **16** (suppl.), 149–165.

BEEBE GW, KATO H AND LAND CE. (1971). Studies of the mortality of A-bomb survivors. IV. Mortality and radiation dose 1950–1960. *Radiat. Res.*, **48**, 613–649.

BITHELL JF. (1989). Epidemiological studies of children irradiated *in utero*. In *Low Dose Radiation: Biological Bases of Risk Assessment*. Baverstock KF, Stather JW, (eds.) pp. 77–87. Taylor and Francis: London.

BITHELL JF AND STEWART AM. (1975). Pre-natal irradiation and childhood malignancy: a review of British data from the Oxford survey. *Br. J. Cancer*, **31**, 271–287.

BLOT WJ, XU Z, BOICE JD, ZHAO D, STONE BJ, SUN J, JING L AND FRAUMENI JF. (1990). Indoor radon and lung cancer in China. *J. Natl Cancer Inst.*, **82**, 1025–1030.

BOWLES RI. (1896). Pathological and therapeutic value of the Roentgen rays. *Lancet*, **1**, 655–656.

BRITISH X-RAY AND RADIUM PROTECTION COMMITTEE. (1921). Preliminary report. *J. Röntgen Soc.*, **17**, 100–103.

BRUES A. (1960). Critique of mutational theories of carcinogenesis. *Acta Union Internationale contre le Cancer*, **16**, 415–417.

BURROWS, EH. (1986). *Pioneers and Early Years: a History of British Radiology*. Colophon: Alderney.

BUTCHER WD. (1905). Protection in X-ray work. *Arch. Roentgen Ray*, **10**, 38–39.

CADE S. (1957). Radiation induced cancer in Man. *Br. J. Radiol.*, **30**, 393–402.

CANNAN RK. (1962). The Atomic Bomb Casualty Commission: the first fourteen years. *News Report (National Academy of Sciences/ National Research Council)*, **12**, 1–7.

CARDIS E, GILBERT ES, CARPENTER L, HOWE G, KATO I, ARMSTRONG BK, BERAL V, COWPER G, DOUGLAS A, FIX J, FRY SA, KALDOR J, LAVE C, SALMON L, SMITH PG, VOELZ GL AND WIGGS LD. (1995). Effects of low doses and low dose rates of external ionizing radiation: cancer mortality among nuclear industry workers in three countries. *Radiation Res.*, **142**, 117–132.

COGAN DG, DONALDSON DD AND REESE AB. (1952). Clinical and pathological characteristics of radiation cataract. *Arch. Ophthalmol.*, **47**, 55–70.

COLWELL HA AND RUSS S. (1934). *X-Ray and Radium Injuries*. Oxford University Press: London.

COMMITTEE FOR THE COMPILATION OF MATERIALS ON DAMAGE CAUSED BY THE ATOMIC BOMBS IN HIROSHIMA AND NAGASAKI. (1981). In *Hiroshima and Nagasaki: the Physical, Medical and Social Effects of the Atomic Bombings*. pp. xiv and 706. Iwanami; Shoter: Tokyo.

COURT BROWN WM AND DOLL R. (1957). Leukaemia and aplastic anaemia in patients irradiated for ankylosing spondylitis. *Med. Res. Council Special Report Series No. 295*. HMSO; London.

DANIEL J. (1896). The X-rays. *Science*, (n.s.) **3**, 526–563.

DARBY SC, OLSEN JH, DOLL R, THAKRAR B, BROWN P deN, STORM HH, BARLOW L, LANGMARK F, TEPPO L AND TULINIUS H. (1992). Trends in childhood leukaemia in the Nordic countries in relation to fallout from atmospheric nuclear weapons testing. *Br. Med. J.*, **304**, 1005–1009.

DARBY SC, WHITLEY E, HOWE GR, HUTCHINGS SJ, KUSIAK RA, LUBIN JH, MORRISON HI, TIRMARCHE M, TOMASEK L, RADFORD EP, ROSCOE RJ, SAMET JM AND SHU XY. (1995). Radon and cancers other than lung cancer in underground miners: a collaborative analysis of 11 studies. *J. Natl Cancer Inst.*, **87**, 378–383.

DOLL R AND WAKEFORD R. (1995). Risk of cancer from fetal irradiation (in preparation).

DOLL R, EVANS HJ AND DARBY SC. (1994). Paternal exposure not to blame. *Nature*, **367**, 678–680.

DUNN K, YOSHIMARU H, OTAKE M, ANNEGERS JF, AND SCHULL WJ. (1990). Prenatal exposure to ionizing radiation and subsequent development of seizures. *Amer. J. Epidemiol.*, **13**, 114–123.

FOLLEY JH, BORGES W AND YAMASAKI T. (1952). Incidence of leukaemia in survivors of the atomic bomb in Hiroshima and Nagasaki. *Am. J. Med.*, **13**, 311–321.

FRANCIS T JR, JABLON S AND MOORE FE. (1955). *Report of ad hoc committee for appraisal of ABCC program*. Technical Report, 33–59. Atomic Bomb Casualty Commission, Hiroshima and Nagasaki.

FRIEBEN A. (1902). Demonstration eines Cancroids des rechten Handriickens, das dich nach langdauernder Einwirkung von Röntgenstrahlen entwickelt hatte. *Fortschr Geb Röntgenstr*, **6**, 106.

FURTH J. (1934). Studies on effects of roentgen rays on lymphomatosis of mice. *Am. J. Roentgenol.*, **32**, 377–383.

GARDNER MJ, SNEE MP, HALL AJ, POWELL CA, DOWNES S AND TERRELL JD. (1990). Results of case–control study of leukaemia and lymphoma among young people near Sellafield nuclear plant in West Cumbria. *Br. Med. J.*, **300**, 423–429.

GLÜCKSMANN A. (1952). Tumour induction by penetrating radiations. In *Biological Hazards of Atomic Energy*. Haddow A. (ed) pp. 87–92. Clarendon Press: Oxford.

HÄRTING FH AND HESSE W. (1879). Der Lungenkrebs, die Bergkrankheit in den Schneeburger Gruben. *Vjschr Gerichtl Med NS*, **31**, 102 and 313.

HAWKINS MM, DRAPER GJ AND WINTER DL. (1995). Cancer in the offspring of survivors of childhood leukaemia and non-Hodgkin's lymphoma. *Br. J. Cancer*, **71**, 1335–1339.

HEWITT D, SANDERS B AND STEWART A. (1966). Progress report IV. Reliability of data reported by case and control mothers. *Monthly Bull Ministry of Health and Public Health Laboratory Service*, **25**, 80–85.

HOFFMAN FL. (1925). Radium (mesothorium) necrosis. *JAMA*, **85**, 961–965.

HOLTHUSEN H, MEYER H AND MOLINEUX W. (1959). Ehrenbuch der Röntgenologen und Radiologen aller Nationen. 2nd edn. *Strahlentherapie*, Suppl. 42.

INTERNATIONAL COMMISSION ON RADIOLOGICAL PROTECTION. (1990). *Recommendations of the International Commission on Radiological Protection*. Publication 60. Pergamon Press: Oxford.

INTERNATIONAL X-RAY AND RADIUM PROTECTION COMMITTEE. (1928). International recommendations for x-ray and radium protection. *Br. J. Radiol.*, **1**, 358–363.

JABLON S, ISHIDA M AND YAMASAKI M. (1965). Studies of the mortality of A-bomb survivors. 3. Description of the sample and mortality, 1950–1960. *Radiat. Res.*, **25**, 25–52.

JAGIC N VON, SCHWARTZ G AND SIEBENROCK L. (1911). Blutbefunde bei Röntgenologen. *Berl Klin Wochenshr*, **48**, 1220–1222.

KATO H. (1971). Mortality in children exposed to A-bombs while *in utero*. *Am. J. Epidemiol.*, **93**, 435–442.

KIENBÖCK R. (1900). Die Einwirkung des Röntgenlichter auf die Haut. *München medizin Wochenschr*, **47**, 1581–1582.

KINLEN LJ, CLARKE K AND BALKWILL A. (1993). Paternal preconceptional radiation exposure in the nuclear industry and leukaemia and non-Hodgkin's lymphoma in young people in Scotland. *Br. Med. J.*, **306**, 1153–1158.

LÉTOURNEAU EG, KREWSKI D, CHOI NW, GODDARD MJ, MCGREGOR RG, ZIELINSKI JM AND DU J. (1994). Case–control study of residential radon and lung cancer in Winnipeg, Manitoba, Canada. *Am. J. Epidemiol.*, **140**, 310–322.

LEWIS EB. (1957). Leukaemia and ionizing radiation. *Science*, **125**, 965–972.

LORENZ E. (1944). Radioactivity and lung cancer; a critical review of lung cancer in the miners of Schneeberg and Joachimsthal. *J. Natl Cancer Inst.*, **5**, 1–15.

LUBIN JH, BOICE JD, EDLING C, HORNUNG RW, HOWE GR, KUNZ E, KUSIAK RA, MORRISON HI, RADFORD EP, SAMET JM, TIRMARCHE M, WOODWARD A, SHU XY AND PIERCE DA. (1995). Lung cancer in radon-exposed miners and estimation of risk from indoor exposure. *J. Natl Cancer Inst.*, **87**, 817–827.

MCCOMBS RS AND MCCOMBS RP. (1930). A hypothesis on the causation of cancer. *Science*, **72**, 423–424.

MCLAUGHLIN JR, KING WD, ANDERSON TW, CLARKE EA AND ASHMORE JP. (1993). Paternal radiation exposure and leukaemia in offspring: the Ontario case–control study. *Br. Med. J.*, **307**, 959–965.

MARCH HC. (1944). Leukaemia in radiologists. *Radiology*, **43**, 275–278.

MARTLAND HS. (1931). The occurrence of malignancy in radioactive persons. *Am. J. Cancer*, **15**, 2435–2516.

MEDICAL RESEARCH COUNCIL. (1956). *The Hazards to Man of Nuclear and Allied Radiations*. HMSO: London.

MEYER H. (1937). Ehrenbuch der Röntgenologen und Radiologen aller National. *Strahlentherapie*, Suppl, 22.

MOLE RH. (1990). Childhood cancer after prenatal exposure to diagnostic X-ray examination in Britain. *Br. J. Cancer*, **62**, 152–168.

MONSON RR AND MACMAHON B. (1984). Prenatal x-ray exposure and cancer in children. In *Radiation Carcinogenesis: Epidemiology and Biological Significance*. Boice JD, Fraumeni JF (eds.) pp. 97–105. Raven Press: New York.

MUIRHEAD CR, COX R, STATHER JW, MACGIBBON BH, EDWARDS AA AND HAYLOCK RGE. (1993). *Estimates of Late Radiation Risks to the UK Population*. Doc. NRPB **4**(4): 15–157. National Radiological Protection Board: Chilton.

MULLER HJ. (1927). Artificial transmutation of the gene. *Science*, **66**, 84–87.

NATIONAL ACADEMY OF SCIENCES. (1956*a*). *Biologic Effects of Atomic Radiation*. National Academy of Sciences: Washington DC.

NATIONAL ACADEMY OF SCIENCES. (1956*b*). *Report of the Committee on Pathologic Effects of Atomic Radiation*. Publication 452. National Academy of Sciences: Washington DC.

NATIONAL RESEARCH COUNCIL. (1988). *Health Risks of Radon and Other Internally Deposited alpha-emitters*. BEIR IV. National Academy Press: Washington DC.

NATIONAL RESEARCH COUNCIL. (1990). *Health Effects of Exposure to Low Levels of Ionizing Radiation*. BEIR V. National Academy Press: Washington DC.

NATIONAL RESEARCH COUNCIL. (1991). *Comparative Dosimetry of Radon in Mines and Homes*. National Academy Press: Washington DC.

NEEL JV AND SCHULL WJ. (1956). *The Effect of Exposure to the Atomic Bombs on Pregnancy Termination in Hiroshima and Nagasaki*. Publication No. 461. National Academy of Sciences, National Research Council: Washington DC.

NEEL JV, SCHULL WJ, MCDONALD DJ, MORTON NE, KODANI M, TAKESHIMA K, ANDERSON RC, WOOD J, BREWER R, WRIGHT S, YAMAZAKI J, SUSUKI M AND KITAMURA S. (1953). The effect of exposure of the atomic bombs on pregnancy termination in Hiroshima and Nagasaki: preliminary report. *Science*, **118**, 537–541.

NEEL JV, SCHULL WJ, AWA AA, SATOH C, KATO H, OTAKE M AND YOSHIMOTO Y. (1990). The children of parents exposed to atomic bombs: estimates of the genetic doubling doses of radiation for humans. *Am. J. Hum. Genet.*, **46**, 1053–1072.

NIELSEN J. (1932). Chronic occupational ray poisoning: discussion based on case of leukaemia in radium worker. *Acta Radiol.*, **13**, 385–394.

OTAKE M AND SCHULL WJ. (1993). Radiation-related small head sizes among prenatally exposed A-bomb survivors. *Int. J. Radiat. Biol.*, **63**, 255–270.

PARKER L, CRAFT AW, SMITH J, DICKINSON H, WAKEFORD R, BINKS K, MCELVENEY D, SCOTT L AND SLOVAK A. (1993). Geographical distribution of preconceptional radiation doses of fathers employed at the Sellafield nuclear installation, West Cumbria. *Br. Med. J.*, **307**, 966–971.

PERSHAGEN G, LIANG Z-H, HRUBEC Z, SVENSSON C AND BOICE JD. (1992). Residential radon exposure and lung cancer in Swedish women. *Health Phys.*, **63**, 179–186.

PERSHAGEN G, ÅKERBLOM G, AXELSON O, CLAVENSJÖ B, DAMBER L, DESAI G, ENFLO A, LAGARDE F, MELLANDER H, SVARTENGREN M AND SWEDJEMARK GA. (1994). Residential radon exposure and lung cancer in Sweden. *New Engl. J. Med.*, **330**, 159–164.

PIAO CO, MARINU SA AND HEI TK. (1990). Radiation, tobacco smoke condensate and oncogenic transformation. 38th Annual Meeting of Radiation Research Society, New Orleans, Abstract. Radiation Research Society, Washington DC.

PIERCE DA AND VAETH M. (1989). Cancer risk estimation from A-bomb survivors: extrapolation to low doses, use of relative risk models and other certainties. In *Low Dose Radiation: Biological Bases of Risk Assessment*. Baverstock KF, Stather JW (eds.) pp. 54–69. Taylor and Francis: London.

PIERCE DA AND VAETH M. (1991). The shape of the cancer mortality dose-response curve for atomic bomb survivors. *Radiat. Res.*, **126**, 36–42.

PIERCE DA, STRAM DO AND VAETH M. (1990). Allowing for random errors in radiation exposures estimates for the atomic bomb survivor data. *Radiat. Res.*, **123**, 275–284.

PLUMMER GW. (1952). Anomalies occurring in children exposed in utero to the atomic bomb in Hiroshima. *Paediatrics*, **10**, 687–693.

RAJEWSKY B. (1939). Bericht über die im Schneeberger Gebiet auf Veranlassung des Reichsausschusses für Krebsbekämpfung durchgeführten Untersuchungen. *Zschr f Krebsforsch*, **47**, 108–111.

ROLLINS WH. (1901). X-light kills. *Boston Med. Surg. J.*, **144**, 173.

ROMAN E, WATSON A, BERAL V, BUCKLE S, BULL D, BAKER K, RYDER H AND BARTON C. (1993). Case–control study of leukaemia and non-Hodgkin's lymphoma among children aged 0–4 years living in West Berkshire and North Hampshire health districts. *Br. Med. J.*, **306**, 615–621.

RÖNTGEN WC. (1895). Ueber eine neue Art von Strahlen. *Sitzungsberichte der Gesellschaft der physik-med. zu Würzburg. pp.* 132–141.

ROSTOSKI O, SAUPE E AND SCHMORL G. (1926). Die Bergkrankheit der Erzbergleute in Schneeberg in Sachsen ('Schneeberger Lungenkrebs'). *Zschr f Krebsforsch*, **23**, 360–384.

RUOSTEENOJA E. (1991). *Indoor Radon and Risk of Lung Cancer: an Epidemiological Study in Finland*. Doctoral dissertation, Department of Public Health, University of Tampere, Finnish Government Printing Office, Helsinki.

SCHOENBERG JB, KLOTZ JB, WILCOX HB, NICHOLLS GP, GIL-DEL-REAL MT, STEMHAGEN A AND MASON TJ. (1990). Case–control study of residential radon and lung cancer among New Jersey women. *Cancer Res.*, **50**, 6520–6524.

SCHULL WJ, NEEL JV AND HASHIZUME A. (1966). Some further observations on the sex ratio among infants born to survivors of the atomic bombings of Hiroshima and Nagasaki. *Am. J. Hum. Genet.*, **18**, 328–338.

SCHULL WJ, OTAKE M AND YOSHIMARU H. (1988). *Effect on Intelligence Test Scores of Prenatal Exposure to Ionizing Radiation in Hiroshima and Nagasaki: a Comparison of the T65DR and DS86 Dosimetry Systems. RERF Tr/3-88*. Radiation Effects Research Foundation: Hiroshima.

SELTSER R AND SARTWELL PE. (1965). The effect of occupational exposure to radiation on the mortality of physicians. *JAMA*, **190**, 1046–1048.

SHIMIZU Y, KATO H, SCHULL WJ AND HOEL DG. (1991). *Life Span Study Report II, Part 3. Non-cancer Mortality, 1950–85, Based on the Revised Doses*. RERF Technical Report Series 2-91. Radiation Effects Research Foundation: Hiroshima.

SMITH PG AND DOLL R. (1981). Mortality from cancer and all causes among British radiologists. *Br. J. Radiol.*, **54**, 187–194.

SPOSTO R, PRESTON DL, SHIMIZU Y AND MABUCHI K. (1992). The effect of diagnostic misclassification on non-cancer and cancer mortality dose–response in A-bomb survivors. *Biometrics*, **48**, 605–617.

STEVENS LG. (1896). Injurious effects on the skin. *Br. Med. J.*, **1**, 998.

STEVENS W, THOMAS DC, LYON JL, TILL JE, KERBER RA, SIMON SL, LLOYD RD, ELGHANY NA AND PRESTON-MARTIN S. (1990). Leukaemia in Utah and radioactive fallout from the Nevada test site: a case–control study. *JAMA*, **264**, 585–591.

STORER JB AND SANDERS PC (1958). Relative effectiveness of neutrons for production of delayed biological effects. *Radiat. Res.*, **8**, 64–70.

STRAUME, T. EGBERT SD. WOULSON WA, FINKEL RC, KUBIK PW, GOVE HE AND HOSHI M. (1992). Neutron discrepancies in the DS86 Hiroshima dosimetry system. *Health Phys.*, **63**, 421–426.

THOMPSON DE, MABUCHI K, RON E, SODA M, TOKUNAGA M, OCHIKUBO S, SUGIMOTO S, IKEDA T, TERASAKI M, IZUME S AND PRESTON DL. (1994). Cancer incidence in atomic bomb survivors. Part II. Solid tumours, 1958–1987. *Radiat. Res.*, **137**, S17–S67.

THOMPSON S. (1897). Presidential address to the Röntgen Society, 5 November 1897. *Arch. Röntgen Ray*, **2**, 23–31.

UHLIG M. (1921). Über den Schneeberger Lungenkrebs. *Virchows Arch.*, **230**, 76–

UNITED NATIONS SCIENTIFIC COMMITTEE ON THE EFFECTS OF ATOMIC RADIATION (1958). *General Assembly Official Records: Thirteenth Session Supplement*. United Nations: New York.

UNITED NATIONS SCIENTIFIC COMMITTEE ON THE EFFECTS OF ATOMIC RADIATION. (1993). *Sources and Effects of Ionizing Radiation. 1993 Report to the General Assembly*. United Nations: New York.

UNITED NATIONS SCIENTIFIC COMMITTEE ON THE EFFECTS OF ATOMIC RADIATION. (1994). *Sources and Effects of Ionizing Radiation. 1994 Report to the General Assembly with Scientific Annexes*. United Nations: New York.

WALSH D. (1897). Deep tissue traumatism from Roentgen ray exposure. *Br. Med. J.*, **2**, 272–273.

WARREN S. (1956). Longevity and causes of death from irradiation in physicians. *JAMA*, **162**, 464–468.

WEISS HA, DARBY SC AND DOLL R. (1994). Cancer mortality following x-ray treatment for ankylosing spondylitis. *Int. J. Cancer*, **59**, 327–338.

YOSHIMOTO Y, KATO H AND SCHULL SJ. (1988). Risk of cancer among children exposed *in utero* to A-bomb radiation, 1950–84. *Lancet*, **2**, 665–669.

YOSHIMOTO Y, NEEL JV, SCHULL WJ, KATO H, SODA M, ETO R AND MABUCHI K. (1990). Malignant tumours during the first two decades of life in the offspring of atomic bomb survivors. *Am. J. Hum. Genet.*, **46**, 1041–1052.